# Bibliometric Analysis of Algal-Bacterial Symbiosis in Wastewater Treatment

**DOI:** 10.3390/ijerph16061077

**Published:** 2019-03-26

**Authors:** Yun Qi, Xingyu Chen, Zhan Hu, Chunfeng Song, Yuanlu Cui

**Affiliations:** 1 Tianjin Key Lab of Biomass/Wastes Utilization, School of Environmental Science and Engineering, Tianjin University, Tianjin 300072, China; qiyun@tju.edu.cn (Y.Q.); chenxingyu@tju.edu.cn (X.C.); huzhan@tju.edu.cn (Z.H.); chunfeng.song@tju.edu.cn (C.S.); 2 Research Center of Traditional Chinese Medicine, Tianjin University of Traditional Chinese Medicine, Tianjin 301617, China

**Keywords:** algal-bacterial, symbiosis, collaborative relationship, wastewater, bibliometric

## Abstract

In recent years, the algae-bacteria symbiotic system has played a significant role in the sustainable development of wastewater treatment. With the continuous expansion of research outputs, publications related to wastewater treatment via algal-bacterial consortia appear to be on the rise. Based on SCI-EXPANDED database, this study investigated the research activities and tendencies of algae-bacteria symbiotic wastewater treatment technology by bibliometric method from 1998 to 2017. The results indicated that environmental sciences and ecology was the most productive subject categories, followed by engineering. Bioresource Technology was the most prominent journal in this field with considerable academic influence. China (146), USA (139) and Spain (76) had the largest amount of publications. Among them, USA was in a leading position in international cooperation, with the highest h-index (67) in 79 countries/territories. The cooperation between China and USA was the closest. The cooperative publishing rate of the Chinese Academy of Sciences was 83.33%, but most of them were in cooperation with domestic institutions, while international cooperation was relatively limited. Methane production, biofuel production, and extracellular polymeric substance were future focal frontiers of research, and this field had gradually become a multi-perspective and inter-disciplinary approach combining biological, environmental and energy technologies.

## 1. Introduction

As a limited natural resource, water is an important part of the global ecosystem, as well as human activities. With the continuous improvement of people’s living standards, water pollution caused by nitrogen, phosphorus, heavy metals, antibiotics, and other environmental pollutants will have a serious impact on the ecological environment, in addition to human health and survival even [1]. High-performance wastewater treatment technology is an effective approach to achieve the sustainable utilization of water resources [2], which can alleviate the pressure of water shortage and ensure the safety of drinking water [3].

Conventional physicochemical wastewater treatment processes have shortcomings, such as high operating cost, low removal efficiency, and a high concern on secondary pollution [4]. From the perspective of biotechnology, water treatment through the life activities of microorganisms has a significant effect on maintaining material metabolism and ecological balance [5]. As an autotrophic microorganism widely distributed in the ecosystem, microalgae can absorb the nitrogen, phosphorus, carbohydrates and other components of wastewater to synthesize their required substances and release O_2_ to the surroundings [6,7]. Finally, the microalgae can be harvested as raw materials for value-added products, such as biofuels, animal feeds or health care products [8]. Using sewage to cultivate microalgae can not only reduce the cost of microalgae biotechnology, but also help to realize the recycling of wastewater [9]. However, it is very difficult to screen algae species with satisfied traits like growth rate, biomass productivity, nutrient removal efficiency, and adaptability to variable outdoor environment simultaneously [10]. Fortunately, researchers have found that microalgae and other wastewater-borne microorganisms, such as bacteria, fungi and so on, can form symbiotic consortia, where these microbes affect and even promote each other [11]. In such a system, microalgae can provide O_2_ and nutrients for the growth of microorganisms, while microorganisms provide CO_2_ and growth stimulating factors for microalgae through respiratory metabolism, consume extracellular polymers and other substances produced by microalgae, and decompose dead algae cells [12]. Meanwhile, the decomposition products of microorganisms can be absorbed and utilized by algae [13]. Algae-bacteria symbiotic technology has been proved to be very remarkable in the treatment of various contaminants [14], although the interaction between microalgae and wastewater-borne microorganisms is complicated and vague [15]. In order to get a comprehensive understanding about the application of algal-bacterial symbiosis in wastewater treatment, qualitative and quantitative assessments of the latest scientific publications are required.

Bibliometric was initially proposed by Alan Pritchard in 1969 [16] and it has been broadly used in various fields for the past few years [17,18]. Garrido-Cardenas and colleagues made a bibliometric analysis in the quantity and distribution of publications, the most relevant journals and keywords to determine the evolution trend of microalgae research [19]. Based on the bibliometric method, the characteristics of publication outputs and the performance of countries and institutions were analyzed, and the future research hotspots on microalgae-derived biodiesel through author keyword analysis were also offered in a recent study [20]. The commonly used bibliometric software includes Excel [21], Bibexcel [22], Gephi [23] and CiteSpace [24,25,26], which are analytical tools based on statistics, bibliometrics, complex social networks and knowledge mapping, respectively. In this research, the basic characteristics (document type and language, subject categories and journals) and the specific performances (publication outputs, growth trend, countries, institutions, keywords) were analyzed, and the global trends of the algae-bacteria symbiotic system for wastewater treatment from 1998 to 2017 were also tracked based on bibliometric method.

## 2. Methods

### 2.1. Data Sources

The information of scientific publications was based on the Web of Science Core Collection. To obtain reliable and accurate details on the topic of algal-bacterial symbiosis in wastewater treatment, 940 publications were obtained on 16 September 2018, using (alga* or micro alga* or micro-alga* or microalga* or *alga*-bacteri* consorti*) and (bacteri* or activated sludge) and (wastewater or sewage) as the search query from Science Citation Index Expanded (SCI-EXPANDED) database for the period from 1998 to 2017.

### 2.2. Bibliometric Analysis

Microsoft Office Excel 2007 was applied to analyze the general research performance of the retrieval literature (language, document type, subject category, journal, publishing year, country, institution, author keywords, etc.). In addition, Bibexcel (Version 2016-02-20) was used for frequency analysis, and the co-occurrence matrix of knowledge units was constructed to evaluate the academic cooperation between the most productive institutions or countries/territories. The impact factor (IF) [27] and h-index [28] proposed by Eugene Garfield and Hirsch, respectively, were the most commonly used indicators for evaluating and quantifying the influence of journals, countries or institutions. This paper used IF value collected by Journal Citation Reports in 2017 and the h-index was calculated by Bibexcel.

### 2.3. Visualization Analysis

Gephi 0.9.2 was used to visualize the social network graph of cooperative relationship analysis, and the visualization tool CiteSpace 5.1.R8 was used for co-occurring keywords study. Finally, the research hotspots and frontiers in this field were analyzed by combining social networks, time zone view and burst detection.

## 3. Results and Discussion

### 3.1. The Characteristics of Research Publications

#### 3.1.1. Document Type and Language

From the database, 940 publications related to algal-bacterial symbiosis in wastewater treatment were classified into six document types. “Article” accounted for 90.00% (846 records), followed by “Review” with 7.87% (92 records), and “Proceedings Paper” with 5.45% (74 records). The remaining publications accounted for less than 0.34%, including “Meeting Abstract”, “Book Chapter” and “News Item”.

Of the total records, 99.04% were printed in English, 0.43% in Polish, 0.32% in French, and Chinese and Portuguese accounted for 0.11% respectively.

#### 3.1.2. Subject Categories and Journals

From 1998 to 2017, a total of 940 records retrieved from SCI-EXPANDED database were involved with 40 subject categories. As listed in Table 1, environmental sciences and ecology contributed the most with 434 records, accounting for 46.17% of the total number of publications, followed by engineering (35.11%), reflecting that the related articles focused on using algae and bacteria as principal part to develop related engineering techniques for sewage purification and ecological remediation. More than 100 papers have been published in biotechnology and applied microbiology (278 records), water resources (187 records), energy and fuels (157 records), and agriculture (116 records). In addition, the statistical results showed that the algal-bacterial symbiosis in wastewater treatment also involved the field of chemistry and toxicology. The highly interdisciplinary property and cross-domain expertise made the system more implementable.

The characteristics of journals in this field were analyzed. As shown in Table 2, all the retrieved articles were divided into 264 academic journals, and the top 10 journals contained 47.17% of all publications. Bioresource Technology was the most productive journal with 107 records (11.38%) which had an impact factor (IF) value of 5.81. Water Science and Technology ranked second with 76 articles (8.09%) followed by Water Research (53), Algal Research—Biomass Biofuels and Bioproducts (34), and Journal of Hazardous Materials (26). It was noticeable that Water Research had the highest IF value (7.05) among these 10 journals, ranking 3rd in all records. In addition, 60.00% of the top 10 productive journals performed relatively well on IF value, ranging from 3 to 7. It reflected the considerable academic influence of algae-bacteria symbiotic technology for wastewater treatment.

### 3.2. The Specific Performance of Research Publications

#### 3.2.1. Characteristics of Publication Outputs

**Figure 1:**
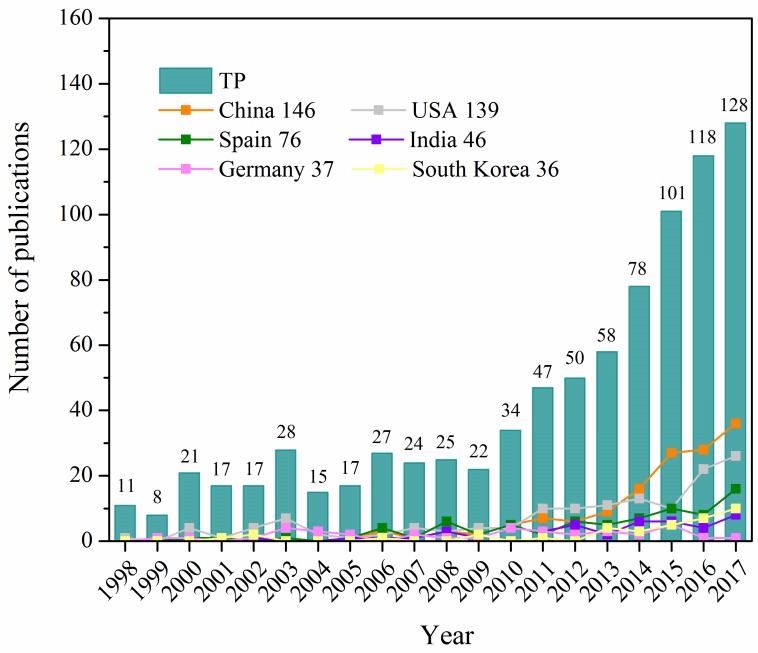
The annual publication number of top 6 productive countries during 1998–2017. TP, the total number of publications. The number after the country is the total number of its publications in this field over the time span.

Since it was the main type of retrieve publications, only articles were analyzed in the subsequent sections. According to Figure 1, the total number of publications on algal-bacterial symbiosis in wastewater treatment from 1998 to 2017 had shown an increasing trend, with slight fluctuations in individual years. Generally, the development of this research can be classified into three stages of evolution. The first stage, from 1998 to 2009, was the basic development stage (initial stage), where the number of annual publications maintained below 30 and the research had just begun to sprout. The second stage was from 2010 to 2014, scholars around the world were paying more attention to the content of algal-bacterial symbiosis in wastewater treatment, and the number of annual publications was between 30 and 80. From that point on, the article was published continuously with a slightly increased growth rate, which was in a relatively rapid development stage (transitional stage). Among them, the publication number in this field increased by 54.50% in 2010 compared with that in 2009. More recently, from 2015 to 2017, the number of publications in the third stage was more than 100 per year, with an annual growth rate of 8.47% to 29.49%. It was in a high-speed development stage (steady stage), of which 128 articles were issued in 2017. In the past 20 years, 846 articles on algal-bacterial symbiosis in wastewater treatment have been published worldwide. The number of publications was not very large, but up to the time of retrieval, data on relevant research papers in the database were still updating with a growth rate which is likely to be further improved. Algal-bacterial symbiosis in wastewater treatment is an emerging topic of research, which enjoys great potential and vast development prospects.

The linear chart in Figure 1 shows the scientific publishing presentation of the 6 most productive countries. In the first stage (1998–2009), the publishing level of all countries around the world was relatively low, while the USA played a leading role with 33 publications in this field. In the second stage (2010–2014), the performance of the USA remained the most prominent country (48 articles). China and Spain kept pace with the global research and continued to develop rapidly, with the number of publications reaching 43 and 25 respectively. Ultimately, after a period of strong growth, China strengthened the global leadership position with 91 publications in the third stage (2015–2017). The number of articles published by China was 1.6 times more than that of the USA, accounting for 26.22% of the total records in this period. The gradual growth in the number of publications might be inseparable from the policies and project deployments issued by governments. China has launched and implemented a number of supporting foundations and projects at the national level, such as the National Natural Science Foundation of China [29,30], the National Program on Key Basic Research Project of China (973 programs) [31], and the National High-Tech Research and Development Program of China (863 programs) [32], etc. Among them, the Major Science and Technology Program for Water Pollution Control and Treatment is one of the 16 major scientific and technological projects established in accordance with the “National medium and long-term plan for science and technology development (2006–2020)”, providing scientific and technological support for water purification and ecological restoration [33]. These projects have effectively promoted the development of wastewater treatment via algal-bacterial symbiosis in China in recent years [34].

#### 3.2.2. Analysis of Growth Trend

As shown in Table 3 [35], the growth trend of relevant characteristics of published articles from 1998 to 2017 was presented. In the first stage (1998–2009), the average annual number of articles was 19, all of which were below 30. During this period, the total number of authors and references were less than 100 and 1000, respectively. Since 2010, the total amount of authors or references of algal-bacterial symbiosis in wastewater treatment led to a remarkable growth. The number of publications increased from 22 in 2009 to 128 in 2017, and the average number of authors per article increased from 3.9 in 1998 to 5.1 in 2017 while the average number of references per article increased from 39.0 to 47.9. As a result, research on algae-bacteria symbiotic systems for wastewater treatment has continued to develop over the past 20 years, and its relations and cooperation are becoming more and more active.

#### 3.2.3. Performance of Countries/Territories

According to the C1 field (research address) and the RP field (address of the corresponding author) derived from SCI-EXPANDED database, the distribution information of countries/territories was analyzed by referring to the method of Ho’s group [36]. Seventy-nine countries/territories in the world conducted relevant research in the field from 1998 to 2017. The top 20 productive countries/territories contributed 76.09% of the total number of published articles (Table 4). China and the USA dominated this research, and their publication records accounted for 17.26% and 16.43% of the total number of publications, respectively. China ranked first in the aspect of the number of total publications, single country’s publications, as well as the publications as first author’s country and corresponding author’s country. However, it had no advantages in terms of the international cooperation and h-index. The total number of publications in the USA was less than that in China, while the cooperation rate and h-index value were quite high. The total number of publications in Spain was about one-half of that in China, but its h-index performance was better. The h-index can be applied to evaluate the quantity and level of academic output effectively, so the USA and Spain probably published higher quality articles than China. Although the relevant research literature in China had some advantages in terms of quantity, it still needed to be improved in quality. The situation in India was similar to that of China in some respects. Its international cooperation and h-index ranked 15th and 13th respectively, but it took 4th place in other information. Compared with the USA, India had a large gap in all aspects. Mexico ranked 9th in total publications, but 4th in the h-index. Although there were no advantages in the total number of publications in Mexico, Italy, the UK and Belgium, their h-index was relatively high.

**Figure 2:**
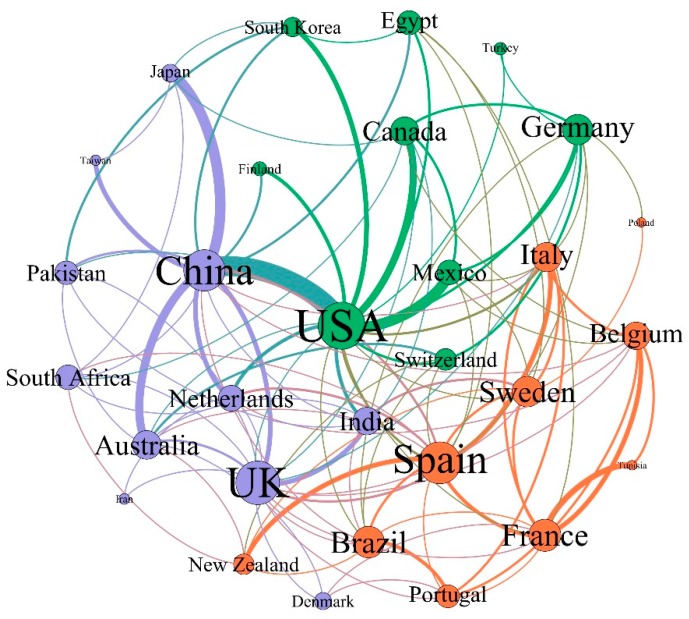
The academic collaborative relationships among the top 30 productive countries/territories.

With the rapid development of economy and the continuous advance of society, international cooperation has become an important form of scientific research [37]. In order to assess the cooperation and activity levels of country/territory in wastewater treatment via algal-bacterial symbiosis system, Gephi was used to conduct social network visualization analysis of data processed by Bibexcel. The academic cooperation between the top 30 countries/territories are shown in Figure 2. Every dot represented a country, the size of the dot indicated the country’s cooperative publishing capability, and the connections between dots revealed the number of cooperative publications with different thicknesses which reflected the cooperative relations among countries. It can be seen that the USA and China were the greatest distributors in the field of algae-bacteria symbiotic wastewater treatment, and the cooperation between these two countries was also the closest, with 17 cooperative publications. The USA displayed the most outstanding performance in international cooperation which was the center of the global collaborative network on this research. Fifty-eight of its 139 published articles were completed in collaboration with the remaining 22 countries. Both China and Spain had international contacts with 25 other countries and have published 55 and 37 articles, respectively. In addition, the cooperation between China and Australia, the Netherlands, Japan and the UK was very close; France and the UK were actively involved in international cooperation, and published fewer articles independently in contrast. Due to the limitations of economic development, India ranked 15th in terms of international cooperation, but performed relatively well in the rest aspects. A total of 50.6% of the countries published more than 3 papers through international cooperation, which indicated that the reuse of wastewater was a global issue for study and discussion, and countries around the world were working together to find better solutions. At the same time, it can be seen in Figure 2 that there was still more room for the development of exchange and cooperation among researchers. For instance, the two major international cooperative countries with the largest publications, the USA and China, have not collaborated with Denmark and Portugal during the past two decades.

#### 3.2.4. Performance of Institutions

Through the analysis of the distribution of the research institution where the author works, we can understand the scientific research capabilities and the explorative atmosphere of the institution. Meanwhile, the institution’s support and recognition of the publications can be reflected from the side. According to the author’s satellite information statistics, research institutions mainly included research universities, high-level research institutions and research centers. From 1998 to 2017, 1044 institutions participated in the research of algal-bacterial symbiosis for wastewater treatment. The top 20 productive institutions were listed in Table 5 with the relevant citation indicators proposed by Ho’s group [38]. Three of them were from the USA, China and Spain, two from New Zealand and one from Belgium, Australia, South Korea, Sweden, Brazil, Germany, the Netherlands, Denmark, and Mexico.

In terms of the property of research institutions, research universities were the mainstay of knowledge innovation, followed by high-level research institutions and research centers. From the perspective of publication numbers, the Chinese Academy of Sciences was the most active, publishing 36 articles, and accounting for 4.26% of the total records, followed by the University of Valladolid in Spain (31), Ghent University in Belgium (19), the University of Leon in Spain (17) and the Harbin Institute of Technology in China (15). The USA and Spain each accounted for 3 of the top 10 productive research institutions, and 2 of them came from China, indicating that these three countries had strong research strength and devoted more in this research, which was consistent with the results of countries/territories distribution analysis. From the point of the h-index, the University of Valladolid ranked first (18), followed by the Chinese Academy of Sciences (16), and the University of California, Berkeley (13). These three research institutions have achieved the most outstanding performance in publication’s impact. Among them, the Chinese Academy of Sciences included many subordinate research units, such as the Research Center for Eco-Environmental Sciences, the Institute of Oceanology, the Institute of Microbiology, and the Institute of Hydrobiology, but its h-index only ranked the second place (Table 5).

Although India, Germany, South Korea, the UK, Italy and Mexico were among the top 10 most productive countries with the largest publications (Table 4), the research institutions in these countries did not appear on the list of the top 10 research institutions (Table 5). The Chinese Academy of Sciences performed best in the total number of publications, independent publications, and cooperative publications. The University of Valladolid in Spain ranked first in terms of the publication as first author’s institution and corresponding author’s institution, with a cooperative rate of 93.55%, while it ranked lower in terms of independent publications.

**Figure 3:**
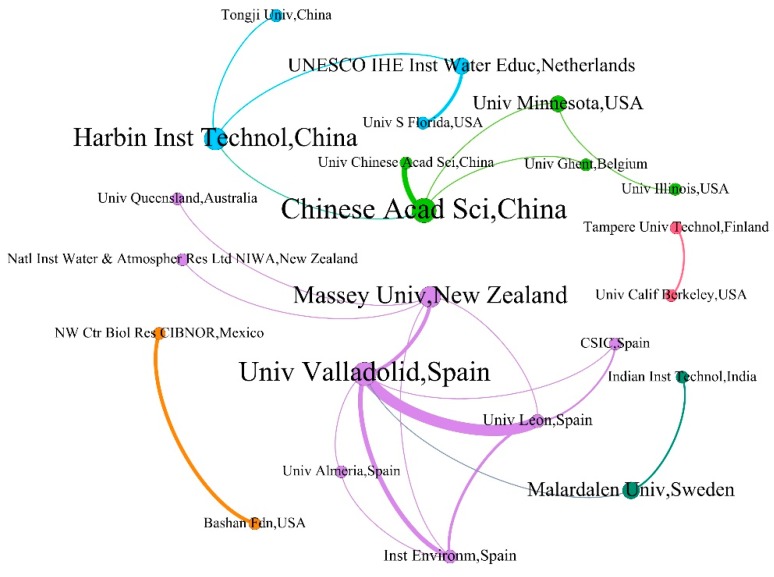
The academic collaborative relationships among the top 30 productive institutions.

In order to investigate the cooperation between research institutions more intuitively, Gephi software was used to visualize data. Figure 3 reflects the collaborative relationship of the top 30 most productive institutions and it can be seen that there were two obvious cooperation networks; one was formed by the Chinese Academy of Sciences, Harbin University of Technology, the University of Minnesota, the University of the Chinese Academy of Sciences, and Gent University. The other was a cooperative network formed by institutions such as the University of Valladolid, the University of Leon, Massey University, Spanish National Research Council and the Spanish Institute of Environment. The Chinese Academy of Sciences had cooperative relations with 45 other research institutes (with a cooperation rate of 83.33%), but most of them were cooperation with Chinese research institutions. Among them, the Chinese Academy of Sciences had the closest contact with the University of Chinese Academy of Sciences (6 articles), while international communication was relatively limited. However, this regional cooperation also significantly promoted China’s scientific production and led to China’s advanced status in terms of algal-bacterial symbiosis for wastewater treatment. In addition, the Northwestern Center for Biological Research (NW Ctr Biol Res) in Mexico and the Bashan Foundation in the USA, the Tampere University of Technology in Finland, and the University of California, Berkeley in the USA only demonstrated cooperation with each other but had no contact with the entire collaboration network. Besides, it should be noted that some institutions could not be found in Figure 3, which means that they have not cooperated with other top 30 productive institutions.

### 3.3. The Main Research Hotspots and Trends

#### 3.3.1. Analysis of Keywords

Keywords are the essence of academic papers [39]. Through the analysis of high-frequency keywords [40], the overall characteristics and development trends of the field can be revealed [41], and research hotspots in this field are able to be obtained more efficiently [42,43]. Using Excel software to analyze the frequency of the author keywords [44], and then 2301 keywords were obtained. From 1998 to 2017, the research on algal-bacterial symbiosis in wastewater treatment was divided into four stages (Table 6). With the passage of time, by observing the changes of the most frequently used author keywords in different time periods, the research emphases and directions can be reflected more directly [36]. *Microalgae*, *wastewater treatment* and *wastewater* were the 3 most frequently encountered keywords. The occurrence frequency of *Chlorella*, *biosorption* and *municipal wastewater* has begun to decline in the last 5 years, showing that research has been deepened and detailed step by step. The frequency of occurrence with *nutrient removal*, *anaerobic digestion*, *ecotoxicity* and *biofuel* has increased gradually, and the combination of biotechnology and wastewater treatment has become more widely available. In addition, *bacteria*, *Chlorella vulgaris*, *activated sludge* and *nitrification* have become hotspots in recent years, and research on algal-bacterial symbiosis in wastewater treatment is gradually developing in this direction. *Nutrient removal* has increased dramatically in the second to third stages. With the continuous development of the social economy, the problem of water eutrophication has drawn public attention. The sudden increase of *anaerobic digestion* and *activated sludge* in the third to fourth stages revealed that these two methods have proved to be new research focus of that period. *Biofilm* and *ecotoxicity* were new keywords appeared in the second stage while *photobioreactors*, *biofuel* and *municipal wastewater* were the research hotspots in the third stage, of which *biofuel* and *ecotoxicity* have received sustained attention in the latter two stages.

#### 3.3.2. Analysis of Research Trends

Co-words analysis is an important method of bibliometrics [45]. By counting the occurrence frequency of a set of keywords in the same article, a co-words network composed of these interrelated keywords can be formed. The length between the nodes in the network can reflect the relationship of the subject content, which in turn demonstrates the structural changes in the research field [46]. In order to objectively analyze the research hotspots of algal-bacterial symbiosis in wastewater treatment, CiteSpace, a kind of citation visualization software, was used to construct the scientific knowledge mapping from two aspects of burst detection and timezone view [47].

##### Burst Detection Analysis

Research frontier is an emerging trend of research theory and subject content, which can be expressed by burst keywords [48]. The burst detection algorithm was proposed by Kleinberg in 2002 and burst keywords refer to words with a suddenly increased relative growth rate in a short period of time [49]. Through the function of burst detection, it is possible to discover the content which does not reach the frequency threshold but have informatics significance in the process of academic development. It can represent the interaction and development trend of the research frontiers more practically and scientifically by detecting the changes in hotspots.

**Figure 4:**
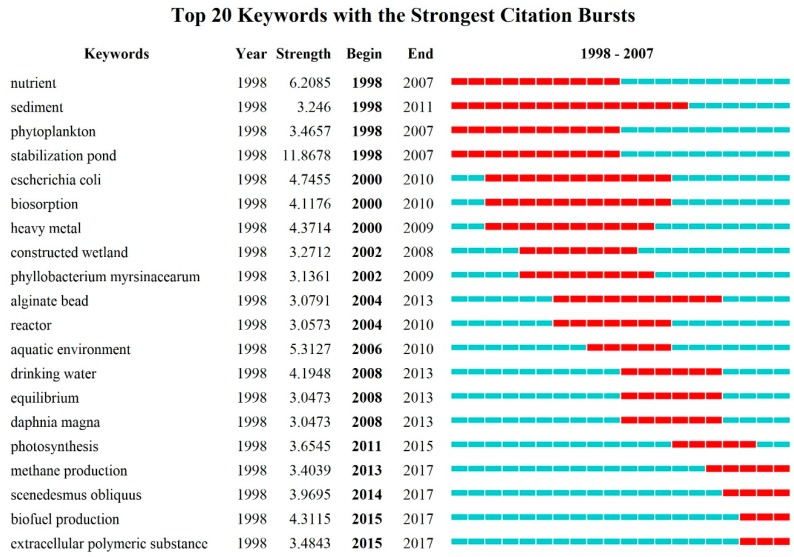
Top 20 keywords with the strongest citation bursts during 1998–2017.

The list of the top 20 keywords with the strongest bursts based on co-occurrence keywords of algal-bacterial symbiosis in wastewater treatment from 1998 to 2017 is shown in Figure 4, which clearly presents the time span and burst strength of the keywords. *Sediment* had a high burst strength 14 years after 1998, while the *nutrient*, *phytoplankton*, and *stabilization pond* experienced a 10-year bursting-out period respectively, and *stabilization pond* had the strongest burst strength (11.87), which means it was a cutting-edge technology between 1998 and 2007. *Escherichia coli*, *biosorption*, *heavy metal*, *constructed wetland*, *Phyllobacterium myrsinacearum*, *aginate bead*, *reactor*, *aquatic environment*, *drinking water*, *equilibrium*, *Daphnia magna*, *Scenedesmus obliquus*, *photosynthesis* and other keywords were breaking out intensively, therefore, the research on algal-bacterial symbiosis in wastewater treatment was no longer just limited to laboratory conditions; it had become an emerging trend to purify water by utilizing the relevant characteristics of dominant algae/bacteria species for suspended and immobilized cultivation. In recent years, the study of algal-bacterial symbiosis in wastewater treatment has been further deepened; *methane production*, *Scenedesmus obliquus*, *biodiesel production* and *extracellular polymeric substance* have become new research frontiers.

In regards to methane production and biodiesel production, the utilization of biomass energy can not only help alleviate the depletion of fossil fuels, but also avoid environmental pollution [50]. Biomass produced by microalgae through photosynthesis can be converted into a variety of renewable energy sources using existing technology [51]. Bioethanol, biodiesel and methane can be produced by fermentation, transesterification, and anaerobic digestion, respectively [52]. Using microalgae-bacteria consortium to treat sewage can increase lipid content and produce methane [53]. Furthermore, the oil accumulated in microalgae cells can be further converted into biodiesel [54].

*Scenedesmus obliquus* is a common green algae in fresh water [55]. It is widely used to treat aquaculture wastewater [56], brewery wastewater [57] and municipal wastewater [58]. Under the stress of nitrogen deficiency, the species can accumulate a large amount of lipids, which can be used as raw material for biodiesel production [59]. Copper-containing wastewater can be effectively treated by the immobilized co-culture system of specific bacteria and *Scenedesmus obliquus* [60].

In biological wastewater treatment systems, extracellular polymeric substance (EPS) is an important high-molecular-weight secretion from microorganisms [61]. It has significant effects on the adsorption, flocculation, sedimentation and dehydration properties of microbial aggregates [62]. In the case of nutrient deficiency, some EPS can be utilized as carbon sources by its own producers [63]. Experiments have proved that EPS can be secreted in algal-bacterial granules systems, which can promote the nutrient removal efficiency, enhance aerobic granulation, improve physico-chemical properties and strengthen system stability [64].

##### Timezone View Analysis

**Figure 5:**
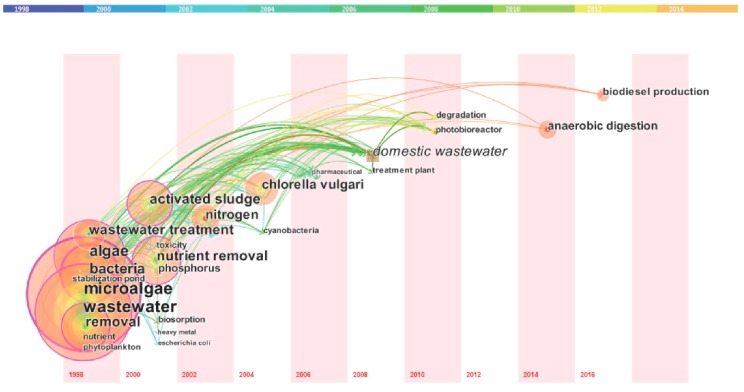
Timezone view based on co-occurring keywords during 1998–2017.

Basic data were imported into CiteSpace software [65], then the timezone view based on the co-occurrence analysis of algal-bacterial symbiosis in wastewater treatment from 1998 to 2017 was obtained (Figure 5) to show the evolution of research hotspots over time.

The length of a unitary time slice was 2 years. Each node corresponded to a keyword in the visualized network, and the location of the node center showed the time when the keyword first appeared in the relevant research; the size of the nodes reflects the occurrence frequency of keywords. The lines between nodes represent the co-occurrence relationship of keywords and the color of lines corresponded to the coherent years shown at the top of the graph. The purple circle shows centrality while the red dot in the nodes represents the burst keywords, indicating the frontier trends in this research. In the primary stage of development, this research focused on microalgae, wastewater, bacteria, nutrient, stabilization pond and other keywords, which were connected with research hotspots in following years. They were the origin of algal-bacterial symbiosis in wastewater treatment, with high centrality and frequency. Subsequent research mainly concentrated on activated sludge, nutrient removal, biosorption, heavy metal, *Escherichia coli*, *Chlorella*, *Cyanobacteria*, pharmaceutical, domestic wastewater, treatment plant, and so on. From 2010 to 2017, the research focuses gradually turned to photobioreactor, degradation, anaerobic digestion and biodiesel production. The research content was more in-depth and specific with the combination of wastewater treatment and bioenergy technology, which had a wide developing prospect.

Figure 4 and Figure 5 show the evolutionary path of the global research front of algal-bacterial symbiosis in wastewater treatment from 1998 to 2017, reflecting the changing of the research topics in different periods. The analysis method based on high-frequency keywords and burst keywords have different emphases. The former shows the research hotspots by the frequency of keywords; and burst detection is taken on the grounds of the gradient of keywords in chronological order, focusing on the development of the keywords themselves. Generally speaking, the stronger the burst strength, the more possible the topic is to become an emerging research trend; the higher the frequency, the more likely it is to become research priorities in this field. On one hand, the high-frequency keywords with low burst strength (such as microalgae, wastewater, and activated sludge in Figure 5) were classic terms that were continuously cited and tended to be stable. On the other hand, keywords with high burst strength (4.31) but low frequency like biodiesel production (Figure 4) attracted more attention and thus may become an emerging research trend in this field.

The algal-bacterial symbiosis in wastewater treatment is an interdisciplinary approach that has grown considerably, which concentrates on biological, environmental and energy science and engineering. But most of the studies related to algal-bacterial symbiosis in wastewater treatment have been carried out in laboratory scale, so it is necessary to optimize relevant parameters in large-scale practical applications in the future research for greater efficiency.

## 4. Conclusions

In this research, the characteristics of publications related to algal-bacterial symbiosis in wastewater treatment from 1998 to 2017 were analyzed, and research hotspots and research frontiers were provided.

Results showed that this research has drawn wide concern, and the number of published articles has continued to increase rapidly over the past two decades. "Environmental sciences and ecology" and "Bioresource Technology" were the most involved subject category and journal included in this research, respectively. The USA and China were the most significant contributors in the field of algal-bacterial symbiosis in wastewater treatment, and the cooperation between them was also very close. The Chinese Academy of Sciences had the strongest cooperation capacity with plenty of domestic institutions, but overseas connections were limited.

According to the analysis of research trends, the scope of the algal-bacterial symbiosis in wastewater treatment has extended to photobioreactors, degradation, anaerobic digestion and biomass energy in recent years. With the efforts of scholars from all over the world, this body of research has been gradually improved and deepened. It has gradually evolved from the basic and unitary subject to a multi-perspective and sustainable development research field combining biology, environmental and energy technology, and thus more interdisciplinary characteristics were reflected. By exploring the characteristics of dominant algae species and bacteria strains, it is going to be an applicable research trend to construct co-culture systems in various methods such as suspension and immobilization. In addition, the incorporation of bioenergy technology and algal-bacterial symbiosis has become an emerging research frontier, which can produce biomass energy such as methane and biodiesel while achieving water purification. It is also considered to be a promising way forward for future research: Establishing energy-saving and environment-friendly technology, making full use of the advantages of metabolic secretions such as EPS in the algal-bacterial symbiosis system, optimizing relevant parameters to improve the efficiency of wastewater treatment, and realizing the comprehensive utilization of renewable resources of microalgae.

## Figures and Tables

**Table 1:** The 10 most productive subjects during 1998–2017.

Rank	Subject	TP	Percentage (%)
1	Environmental sciences and ecology	434	46.17
2	Engineering	330	35.11
3	Biotechnology and applied microbiology	278	29.57
4	Water resources	187	19.89
5	Energy and fuels	157	16.70
6	Agriculture	116	12.34
7	Marine and freshwater biology	85	9.04
8	Chemistry	78	8.30
9	Microbiology	48	5.11
10	Toxicology	39	4.15

TP, the number of total publications.

**Table 2:** The 10 most productive journals during 1998–2017.

Rank	Journal	TP	Percentage (%)	IF 2017
1	Bioresource Technology	107	11.38	5.81
2	Water Science and Technology	76	8.09	1.25
3	Water Research	53	5.64	7.05
4	Algal Research—Biomass Biofuels and Bioproducts	34	3.62	3.75
5	Journal of Hazardous Materials	26	2.77	6.43
6	Ecological Engineering	25	2.66	3.02
7	Journal of Applied Phycology	18	1.92	2.40
8	Environmental Toxicology and Chemistry	17	1.81	3.18
9	Desalination and Water Treatment	16	1.70	1.38
10	Environmental Science and Pollution Research	15	1.60	2.80

TP, the number of total publications.

**Table 3:** Characteristics of periodical articles from 1998 to 2017.

PY	TP	No.AU	AU/TP	NR	NR/TP	PG	PG/TP
1998	11	43	3.91	429	39.00	135	12.27
1999	8	33	4.13	232	29.00	93	11.63
2000	21	83	3.95	548	26.10	200	9.52
2001	17	63	3.71	533	31.35	131	7.71
2002	17	57	3.35	545	32.06	177	10.41
2003	28	90	3.21	769	27.46	282	10.07
2004	15	57	3.80	423	28.20	152	10.13
2005	17	73	4.29	381	22.41	151	8.88
2006	27	104	3.85	892	33.04	251	9.30
2007	24	97	4.04	732	30.50	232	9.67
2008	25	103	4.12	1043	41.72	223	8.92
2009	22	87	3.95	933	42.41	214	9.73
2010	34	161	4.74	1307	38.44	315	9.26
2011	47	199	4.23	1768	37.62	392	8.34
2012	50	222	4.44	1861	37.22	457	9.14
2013	58	303	5.22	2328	40.14	529	9.12
2014	78	382	4.90	2875	36.86	715	9.17
2015	101	547	5.42	4180	41.39	911	9.02
2016	118	580	4.92	5219	44.23	1077	9.13
2017	128	659	5.15	6134	47.92	1309	10.23

PY, Year; TP, the total number of publications; No.AU, the total number of authors; AU/TP, average number of authors per article; NR, the total number of references; NR/TP, average number of references per article; PG, the number of pages; PG/TP, average number of pages per article.

**Table 4:** Top 20 productive countries/territories during 1998–2017.

Country	TP	TP R (%)	SP R (%)	CP R (%)	FP R (%)	RP R (%)	C	C (%)	R (h-Index)
China	146	1 (17.28)	1 (14.61)	2 (24.77)	1 (14.91)	1 (14.93)	55	37.67	3 (21)
USA	139	2 (16.45)	2 (13.00)	1 (26.13)	2 (12.07)	2 (12.09)	58	41.73	1 (67)
Spain	76	3 (8.99)	3 (6.26)	3 (16.67)	3 (8.05)	3 (8.06)	37	48.68	2 (23)
India	46	4 (5.44)	4 (5.78)	15 (4.50)	4 (4.62)	4 (4.62)	10	21.74	13 (9)
Germany	37	5 (4.38)	6 (3.85)	10 (5.86)	8 (3.08)	8 (3.08)	13	35.14	9 (10)
South Korea	36	6 (4.26)	5 (4.33)	18 (4.05)	5 (3.79)	5 (3.79)	9	25.00	6 (11)
UK	33	7 (3.91)	13 (2.25)	5 (8.56)	10 (2.84)	10 (2.84)	19	57.58	6 (11)
Italy	33	7 (3.91)	8 (3.21)	10 (5.86)	7 (3.20)	7 (3.20)	13	39.39	5 (12)
Mexico	32	9 (3.79)	11 (2.57)	6 (7.21)	6 (3.55)	6 (3.55)	16	50.00	4 (14)
Australia	32	9 (3.79)	9 (2.89)	8 (6.31)	10 (2.84)	10 (2.84)	14	43.75	9 (10)
Belgium	31	11 (3.67)	12 (2.41)	6 (7.21)	9 (2.96)	9 (2.96)	16	51.61	6 (11)
Netherlands	27	12 (3.20)	15 (2.09)	8 (6.31)	16 (1.89)	16 (1.90)	14	51.85	13 (9)
Canada	26	13 (3.08)	13 (2.25)	13 (5.41)	14 (2.13)	14 (2.13)	12	46.15	9 (10)
New Zealand	25	14 (2.96)	9 (2.89)	20 (3.15)	13 (2.49)	13 (2.49)	7	28.00	15 (8)
France	25	14 (2.96)	29 (0.80)	4 (9.01)	21 (1.42)	21 (1.42)	20	80.00	15 (8)
Turkey	24	16 (2.84)	7 (3.53)	41 (0.90)	12 (2.72)	12 (2.73)	2	8.33	19 (5)
Brazil	23	17 (2.72)	15 (2.09)	15 (4.50)	15 (2.01)	15 (2.01)	10	43.48	9 (10)
Japan	22	18 (2.60)	20 (1.44)	10 (5.86)	16 (1.89)	16 (1.90)	13	59.09	15 (8)
Portugal	21	19 (2.49)	17 (1.77)	15 (4.50)	19 (1.54)	19 (1.54)	10	47.62	15 (8)
Sweden	19	20 (2.25)	24 (1.12)	13 (5.41)	18 (1.78)	18 (1.78)	12	63.16	15 (8)

TP, the total number of publications; TP R (%), the ranking and percentage of the total number of articles; SP R (%), the ranking and percentage of the single country’s publication; CP R (%), the ranking and percentage of internationally collaborative publications; FP R (%), the ranking and percentage of articles for the country as the first author’s country; RP R (%), the ranking and percentage of articles for the country as the corresponding author’s country; C, the number of articles published by the country in cooperation with other countries; C%, the percentage of articles published by the country in cooperation with other countries of the country’s total publications.

**Table 5:** Top 20 productive institutions during 1998–2017.

Institution	TP	TP R (%)	SP R (%)	CP R (%)	FP R (%)	RP R (%)	C	C (%)	R (h-Index)
Chinese Acad Sci, China	36	1 (4.26)	1 (1.75)	1 (5.96)	2 (2.72)	2 (2.73)	30	83.33	2 (16)
Univ Valladolid, Spain	31	2 (3.20)	21 (0.58)	2 (5.77)	1 (2.84)	1 (2.84)	29	93.55	1 (18)
Univ Ghent, Belgium	19	3 (2.25)	1 (1.75)	4 (2.58)	3 (2.01)	3 (2.01)	13	68.42	4 (11)
Univ Leon, Spain	17	4 (1.30)	21 (0.58)	3 (2.98)	63 (0.24)	63 (0.24)	15	88.24	5 (9)
Harbin Inst Technol, China	15	5 (1.78)	9 (0.88)	5 (2.39)	4 (0.95)	4 (0.95)	12	80.00	10 (6)
Univ Minnesota, USA	10	6 (1.18)	21 (0.58)	6 (1.59)	22 (0.47)	22 (0.47)	8	80.00	7 (7)
Univ Calif Berkeley, USA	9	7 (1.07)	21 (0.58)	7 (1.39)	13 (0.59)	13 (0.59)	7	77.78	3 (13)
CSIC, Spain	9	7 (1.07)	21 (0.58)	7 (1.39)	4 (0.95)	4 (0.95)	7	77.78	7 (7)
Univ S Florida, USA	8	9 (0.95)	59 (0.29)	7 (1.39)	10 (0.71)	10 (0.71)	7	87.50	10 (6)
Univ Queensland, Australia	8	9 (0.95)	21 (0.58)	14 (1.19)	22 (0.47)	22 (0.47)	6	75.00	14 (5)
Tongji Univ, China	8	9 (0.95)	6 (1.17)	22 (0.80)	6 (0.83)	6 (0.83)	4	50.00	14 (5)
Natl Inst Water & Atmospher Res Ltd NIWA, New Zealand	8	9 (0.95)	6 (1.17)	22 (0.80)	6 (0.83)	6 (0.83)	4	50.00	19 (3)
Malardalen Univ, Sweden	8	9 (0.95)	59 (0.29)	7 (1.39)	6 (0.83)	6 (0.83)	7	87.50	18 (4)
Korea Adv Inst Sci & Technol, South Korea	8	9 (0.95)	59 (0.29)	7 (1.39)	22 (0.47)	22 (0.47)	7	87.50	10 (6)
Univ Fed Vicosa, Brazil	7	15 (0.83)	6 (1.17)	36 (0.60)	10 (0.71)	10 (0.71)	3	42.86	7 (7)
UNESCO IHE Inst Water Educ, Netherlands	7	15 (0.83)	21 (0.58)	19 (0.99)	38 (0.36)	38 (0.36)	5	71.43	10 (6)
UFZ Helmholtz Ctr Environm Res, Germany	7	15 (0.83)	59 (0.29)	14 (1.19)	141 (0.12)	141 (0.12)	6	85.71	14 (5)
Tech Univ Denmark, Denmark	7	15 (0.83)	4 (1.46)	81 (0.40)	13 (0.59)	13 (0.59)	2	28.57	14 (5)
NW Ctr Biol Res CIBNOR, Mexico	7	15 (0.83)	N/A	7 (1.39)	6 (0.83)	6 (0.83)	7	100.00	6 (8)
Massey Univ, New Zealand	7	15 (0.83)	N/A	7 (1.39)	38 (0.36)	38 (0.36)	7	100.00	19 (3)

TP, the total number of publications; TP R (%), the ranking and percentage of the total number of articles; SP R (%), the ranking and percentage of the single institution’s publication; CP R (%), the ranking and percentage of internationally collaborative publications; FP R(%), the ranking and percentage of articles for the institution as the first author’s institution; RP R (%), the ranking and percentage of articles for the institution as the corresponding author’s institution; C, the number of articles published by the institution in cooperation with other institutions; C%, the percentage of articles published by the institution in cooperation with other institutions of the institution’s total publications; N/A, not available.

**Table 6:** Top 20 most frequently used author keywords in different periods during 1998–2017.

Author Keywords	TP	1998–2017 R (%)	1998–2002 R (%)	2003–2007 R (%)	2008–2012 R (%)	2013–2017 R (%)
microalgae	125	1 (16.17)	5 (9.23)	5 (5.38)	1 (9.04)	1 (22.05)
wastewater treatment	90	2 (11.64)	1 (18.46)	2 (8.60)	3 (8.43)	3 (12.47)
wastewater	89	3 (11.51)	4 (10.77)	1 (10.75)	1 (9.04)	2 (12.69)
algae	71	4 (9.18)	3 (12.31)	7 (4.30)	4 (7.83)	4 (10.24)
nutrient removal	39	5 (5.05)	44 (1.54)	21 (2.15)	7 (4.22)	5 (6.46)
bacteria	34	6 (4.40)	2 (13.85)	4 (6.45)	11 (3.01)	9 (3.12)
Chlorella vulgaris	31	7 (4.01)	N/A	10 (3.23)	16 (2.41)	6 (5.35)
Chlorella	25	8 (3.23)	7 (6.15)	10 (3.23)	5 (5.42)	14 (2.00)
anaerobic digestion	25	8 (3.23)	44 (1.54)	N/A	35 (1.20)	7 (4.90)
activated sludge	24	10 (3.10)	11 (4.62)	10 (3.23)	35 (1.20)	8 (3.56)
nutrients	22	11 (2.85)	6 (7.69)	2 (8.60)	35 (1.20)	22 (1.56)
biofilm	22	11 (2.85)	N/A	56 (1.08)	6 (4.82)	10 (2.90)
biosorption	19	13 (2.46)	18 (3.08)	5 (5.38)	11 (3.01)	22 (1.56)
photobioreactor	17	14 (2.20)	N/A	N/A	7 (4.22)	12 (2.23)
nitrification	16	15 (2.07)	7 (6.15)	21 (2.15)	23 (1.81)	22 (1.56)
nitrogen removal	15	16 (1.94)	18 (3.08)	10 (3.23)	35 (1.20)	16 (1.78)
ecotoxicity	15	16 (1.94)	N/A	21 (2.15)	16 (2.41)	14 (2.00)
biofuel	15	16 (1.94)	N/A	N/A	23 (1.81)	11 (2.67)
toxicity	14	19 (1.81)	18 (3.08)	21 (2.15)	23 (1.81)	22 (1.56)
municipal wastewater	14	19 (1.81)	N/A	N/A	9 (3.61)	16 (1.78)

TP, the total number of publications; R (%), ranking and percentage of author keywords; N/A, not available.
